# Correlating genotypic and phenotypic antimicrobial susceptibility testing in clinical *Chlamydia trachomatis* isolates

**DOI:** 10.1128/spectrum.02935-23

**Published:** 2023-11-15

**Authors:** Arabella Touati, Cécile Bébéar, Olivia Peuchant

**Affiliations:** 1 Bacteriology Department, CHU Bordeaux, National Reference Center for Bacterial Sexually Transmitted Infections, Bordeaux, France; 2 Univ. Bordeaux, Centre national de la recherche scientifique (CNRS), UMR 5234, Fundamental Microbiology and Pathogenicity, Bordeaux, France; Ann & Robert H. Lurie Children's Hospital of Chicago, Chicago, Illinois, USA

**Keywords:** *Chlamydia trachomatis*, genotypic resistance, phenotypic susceptibility

## LETTER

Nucleic acid amplification tests are commonly used to detect *Chlamydia trachomatis,* and culture is not routinely carried out to isolate *C. trachomatis* from clinical specimens. Therefore, it is difficult to survey the antimicrobial susceptibility of clinical isolates of *C. trachomatis*. Resistance monitoring could be realized using genotypic markers directly from specimens, but it requires that a correlation between genotype and phenotypic antimicrobial susceptibility testing (AST) has been established beforehand.

We performed AST of 24 *C*. *trachomatis* clinical isolates collected in 2018 at the French National Reference Center for STI. Isolates belonged to genovar E (*n* = 13), D/Da (*n* = 4), F (*n* = 3), G (*n* = 2), K (*n* = 1), and Ia (*n* = 1). Three antibiotics were tested: azithromycin, doxycycline, and ofloxacin. The minimal inhibitory concentration (MIC) was obtained by inoculation of *C. trachomatis* on the McCoy cell line in the presence of increasing concentrations of antibiotics (1). The MIC was defined as the concentration of the drug after the one in which 90% or more of the inclusions were altered in size and morphology (1). The 16S and 23S rRNA, *rpl*D (L4), *rpl*V (L22), and *gyr*A genes were sequenced, and identified mutations were correlated with MICs (2 – 5).

All clinical isolates displayed MICs similar to those from the L2/434/Bu reference strain. Azithromycin MICs ranged between 0.125 and 0.5 µg/mL (Table 1). Molecular analysis did not reveal any mutations in the 23S rRNA except for the L2/434/Bu reference strain which harbored the A2094G mutation (*Escherichia coli* numbering). This mutation, occurring outside the peptidyl transferase loop of domain V of the 23S rRNA, has not yet been associated with macrolide resistance. Two isolates harbored the V29I mutation in the *rpl*V gene, not associated with resistance. Doxycycline MICs ranged from 0.015 to 0.125 µg/mL. Sequencing of the 16S rRNA gene showed that 70.8% (17/24) of the isolates harbored both G1247A and C1257T mutations. Two isolates had one additional mutation each, T627G and A600G, respectively. Mutations in the 16S rRNA gene previously associated with tetracycline resistance in other bacterial species are located in the primary tetracycline-binding site formed by nucleotides 964–967 of helix 31 (H31) and nucleotides 1,054–1,056 and 1,196–1,200 of helix 34 (H34) (6, 7). Such mutations have not been identified in our study. Ofloxacin MICs ranged between 0.5 and 1 µg/mL. The amino acid changes V61A and H129Q in the GyrA protein were found in 79.2% (19/24) of the isolates. Moreover, all the five strains with the wild-type GyrA harbored the silent mutation C237T. These mutations are not included within the quinolone-resistance-determining regions and are not associated with ofloxacin resistance, as previously described (8).

**TABLE 1:** Azithromycin, doxycycline, and ofloxacin MICs for *C. trachomatis* clinical isolates and mutations found in the 16S-23S rRNA genes, ribosomal protein L4 and L22, and GyrA protein

*C. trachomatis* isolate			Azithromycin MIC (µg/ml)	23S rRNA gene^*a*^	Ribosomal protein L4^*b*^	Ribosomal protein L22^*b*^	Doxycycline MIC (µg/mL)	16S rRNA gene^*a*^	Ofloxacin MIC (µg/mL)	GyrA protein^*b*^
Name	Genovar	Origin								
1748	Ia	Cervix	0.25	WT^*c*^	WT	V29I	0.06	WT	1	WT (C237T: silent)
1749	E	Cervix	0.25	WT	WT	WT	0.03	G1247A-C1257T	1	V61A-H129Q
1755	E	Cervix	0.5	WT	WT	WT	0.06	G1247A-C1257T	1	V61A-H129Q
1756	E	Cervix	0.25	WT	WT	WT	0.06	T627G-G1247A-C1257T	1	V61A-H129Q
1758	E	Cervix	0.25	WT	WT	WT	0.06	G1247A-C1257T	0.5	V61A-H129Q
1759	E	Vaginal swab	0.25	WT	WT	WT	0.03	G1247A-C1257T	1	V61A-H129Q
1760	E	Cervix	0.25	WT	WT	WT	0.06	G1247A-C1257T	0.5	V61A-H129Q
1761	D/Da	Vaginal swab	0.25	WT	WT	WT	0.03	G1247A-C1257T	0.5	V61A-H129Q
1762	E	Cervix	0.5	WT	WT	WT	0.06	G1247A-C1257T	1	V61A-H129Q
1764	F	Cervix	0.25	WT	WT	WT	0.03	G1247A-C1257T	1	V61A-H129Q
1765	E	Cervix	0.25	WT	WT	WT	0.06	G1247A-C1257T	0.5	V61A-H129Q
1772	D/Da	Cervix	0.5	WT	WT	WT	0.03	WT	1	WT (C237T: silent)
1770	E	Cervix	0.5	WT	WT	WT	0.06	G1247A-C1257T	0.5	V61A-H129Q
1767	G	Cervix	0.5	WT	WT	V29I	0.06	WT	1	WT (C237T: silent)
1773	E	Cervix	0.25	WT	WT	WT	0.06	G1247A-C1257T	0.5	V61A-H129Q
1774	E	Cervix	0.5	WT	WT	WT	0.03	G1247A-C1257T	1	V61A-H129Q
1775	D/Da	Cervix	0.25	WT	WT	WT	0.03	G1247A-C1257T	0.5	V61A-H129Q
1778	D/Da	Cervix	0.5	WT	WT	WT	0.03	G1247A-C1257T	0.5	V61A-H129Q
1784	E	Urethral	0.5	WT	WT	WT	0.03	G1247A-C1257T	0.5	V61A-H129Q
1785	F	Cervix	0.5	WT	WT	WT	0.03	A600G-G1247A-C1257T	1	V61A-H129Q
1786	K	Cervix	0.25	WT	WT	WT (T139C:silent)	0.06	WT	0.5	WT (C237T: silent)
1790	G	Cervix	0.5	WT	WT	WT	0.125	WT	1	WT (C237T: silent)
1828	F	Cervix	0.25	WT	WT	WT	0.03	G1247A-C1257T	1	V61A-H129Q
1834	E	Cervix	0.5	WT	WT	WT	0.03	G1247A-C1257T	0.5	V61A-H129Q
L2 434/Bu reference strain	L2		0.25	A2094G	WT	WT	0.06	WT	0.5	WT

^*a*^ *E. coli* numbering.

^*b*^ *C*. *trachomatis* L2 numbering.

^*c*^ WT, wild-type.

Monitoring antimicrobial susceptibility is of concern in *C. trachomatis*, especially with the rise in the use of doxycycline as an antibiotic off-label for STI prophylaxis. Doxycycline is currently the recommended first-line treatment for genital chlamydial infection in the United States and the United Kingdom, and to date, no cases of resistance to doxycycline have been reported for *C. trachomatis* (9, 10). Acquired resistance to azithromycin or ofloxacin, the second-line treatment for *C. trachomatis* infection, could lead to the threat of the spread of antibiotic-resistant strains, although no major challenge in antimicrobial resistance has been identified so far. *In silico* antimicrobial resistance predictions for *C. trachomatis* could help to monitor its antimicrobial susceptibility profile and the acquisition of antibiotic resistance. In this study, we showed that none of the identified mutations in *C. trachomatis* have a role in phenotypic antibiotic resistance.
